# Angiokératome acquis de la face

**DOI:** 10.11604/pamj.2018.30.140.14943

**Published:** 2018-06-19

**Authors:** Jawad El-Azhari, Naoufal Hjira

**Affiliations:** 1 Service de Dermatologie-Vénérologie, Hôpital Militaire d’Instruction Mohammed V, Rabat, Maroc

**Keywords:** Angiokératome, face, acquis, électrocoagulation, Angiokeratoma, face, acquired, electrocoagulation

## Abstract

Angiokeratomas are vascular dilations (telangiectasic papules) with keratotic surface caused by capillary dilation in the dermal papilla due to venous hyperpression, vascular malformation or a capillary fragility. They most commonly are benign and just unsightly and they may sometimes reveal a dreaded overload disease known as Fabry Disease. Here we report the case of a 56-year old patient, with no notable medical history, presenting with nontraumatic, nonpruritic, bleeding on contact, grouped cluster erythematous papules without extracutaneous manifestations on the left malar region, that had occurred 4 months before. Diascopy revealed partially emptied papules with keratotic surface. Kaposi syndrome, melanoma, carcinoma or angiokeratoma were suspected. Histological examination revealed angiokeratoma. Treatment based on electrocoagulation was proposed to the patient.

## Image en médecine

Les angiokératomes sont des dilatations vasculaires (papules télangiectasiques) dont la surface est kératosique et qui résultent de la dilatation des capillaires de la papille dermique, due à une hyperpression veineuse, à une malformation vasculaire ou à une fragilité capillaire. Le plus souvent bénins et seulement inesthétiques, ils peuvent parfois révéler une redoutable maladie de surcharge comme la maladie de Fabry. Nous rapportons ici le cas d’un patient de 56 ans, sans antécédents notables, qui consulte pour des papules érythémateuses groupées en bouquet au niveau de la région malaire gauche, non prurigineuses, et saignant au contact, apparues depuis 4 mois sans notion de traumatisme antérieur et sans symptomes extra-cutanés. A l’examen les papules étaient partiellement vidées à la vitropression et la surface était kératosique. Les diagnostics évoqués étaient un kaposi, un mélanome, un carcinome ou un angiokeratome. L’histologie était en faveur d’un angiokeratome, et le traitement proposé était l’électrocoagulation.

**Figure 1:**
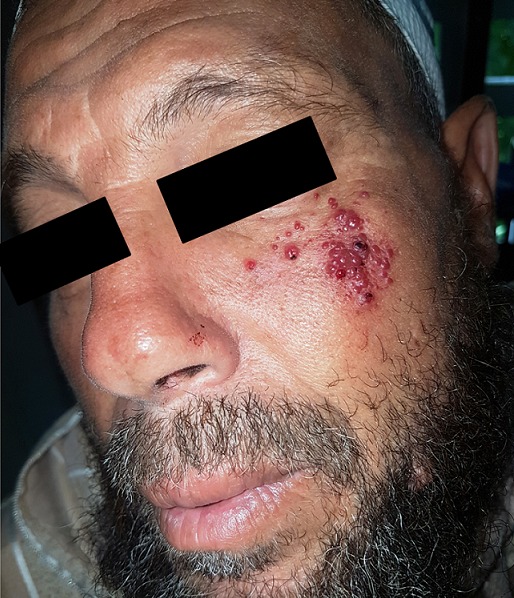
angiokératome malaire gauche

